# Whole Genome Resequencing Reveals Selection Signatures Associated With Important Traits in Ethiopian Indigenous Goat Populations

**DOI:** 10.3389/fgene.2019.01190

**Published:** 2019-11-28

**Authors:** Haile Berihulay, Yefang Li, Berihu Gebrekidan, Gebremedhin Gebreselassie, Xuexue Liu, Lin Jiang, Yuehui Ma

**Affiliations:** ^1^Institute of Animal Science, Chinese Academy of Agricultural Sciences, Beijing, China; ^2^College of Veterinary Science, Mekelle University, Mekelle, Ethiopia

**Keywords:** candidate genes, *Capra hircus*, pooled heterozygosity, population differentiation, positive selection signature

## Abstract

Ethiopia is considered as the main gateway for the introduction of livestock species, including goat, to the African continent. Ethiopian goats are characterized by their unique adaptive ability, and different physical characteristics in terms of morphology, body size, coat colors, and other important traits. The comparative population genomic analysis provides useful genomic information associated with important traits. Whole-genome resequencing of 44 Ethiopian indigenous goats produced 16 million single-nucleotide polymorphisms (SNPs) as well as 123,577 insertions and deletions. Specifically, 11,137,576, 10,760,581, 10,833,847, 12,229,657 and 10,749,996 putative SNPs were detected in Abergelle, Afar, Begait, Central Highland and Meafure goat populations, respectively. In this study, we used population differentiation (*F*_ST_) and pooled heterozygosity (*H_P_*) Cbased approaches. From the *F*_ST_ analysis, we identified 480 outlier windows. The *H_P_* approach detected 108 and 205 outlier windows for Abergelle, and Begait, respectively. About 11 and 5 genes under selective signals were common for both approaches that were associated with important traits. After genome annotation, we found 41 Gene ontology (GO) terms (12 in biological processes, 8 in cellular components and 11 in the molecular function) and 10 Kyoto Encyclopedia of Genes and Genomes pathways. Several of the candidate genes are involved in the reproduction, body weight, fatty acids, and disease related traits. Our investigation contributes to deliver valuable genetic information and paves the way to design conservation strategy, breed management, genetic improvement, and utilization programs. The genomic resources generated in the study will offer an opportunity for further investigations.

## Introduction

Goats (*Capra hircus*) are one of the first ruminant animals to be domesticated around 10,000 to 9900 years ago (Zeder and Hesse, 2000) at the dawn of the Neolithic period in the Fertile Crescent. They were subsequently dispersed across the continents, adapting themselves to diverse biophysical and production environments. This also holds true for Ethiopia, which is considered as the main gateway for the introduction of livestock species, including goat, to the African continent (Gifford-Gonzalez and Hanotte, 2011). The country is home for 30.2 million goats (CSA, 2017), with seven genetically characterized groups and 14 local phenotypically classified populations (Getinet, 2016) which are characterized by their unique adaptive ability, and different physical characteristics in terms of morphology, body size, coat colors, and other production traits (Hassen et al., 2012), this implies that natural selection has most probably played a key role in the adaptation of these populations under the diverse range of environments. They are also known as the livelihood of resource-poor farmers and pastoralists, because of their immense economic importance trait, substantial phenotypic diversity and evolved in harsh climatic conditions (Hassen et al., 2012). Ethiopian indigenous goats are primarily used as a source of income, milk, meat, manure and many other sociocultural functions (Abegaz et al., 2013).

Among the Ethiopian goat populations, Begait and Afar are well adapted to the hotter and drier low land areas with a high temperature (27 to 37°C) and low rainfall ranging from 250 to 747 mm/years (Abegaz et al., 2013), and they are likely under selection for thermal stress, while the Abergelle, Central highland, and Meafure goats are mainly distributed from mid to mountainous production environments characterized by high rainfall (650 up to 700 mm) and relatively low temperature (5-30°C) (Zerabruk and Vangen, 2005). Moreover, Begait goat can be identified by their tall, predominantly white coat color and hairy thighs with long drooping ears (Berhane and Eik, 2006). They are merits of tolerance to feed and water shortages, resistance to diseases, and have the ability to produce and reproduce under such conditions and therefore, their genomes should harbor footprints for selection program for multi-traits.

Hence, genomic characterization of the Begait goat breed using modern genomic tools is paramount important to ensure breeding of these hardy goat populations. In this study, we reported genes that are positively selected in Begait goat population associated with important traits by scanning the whole genome resequencing data. The availability of whole genome resequencing data has facilitated the mapping of signatures selection associated with domestication and selection (Lai et al., 2016; Guo et al., 2018). Several investigations of whole genome resequencing have previously been examined genetic divergence among populations, identify potential genes/genomic regions associated with production, reproduction and adaption traits in various livestock species including goats (Benjelloun et al., 2015; Wang et al., 2016; Guo et al., 2018), sheep (Yang et al., 2016), cattle (Taye et al., 2017), pig (Li et al., 2014).

In Africa, studies on goat have been reported to detect positive selection signatures mainly focused with environmental adaptation and thermo-tolerance under heat stress conditions (Kim et al., 2016; Onzima et al., 2018). However, there have been no previous studies conducting in important traits in Ethiopian goat populations. Here we reported a positive selection signature of goat populations that is associated with important traits using whole-genome resequencing of five Ethiopian goat populations.

In this study, we applied a population differentiation (Akey et al., 2010) and pooled heterozygosity (Rubin et al., 2010) based approaches to explore the genetic mechanisms underlying variation across the whole genome resequence and are an actual approach to detect selection signature for populations with unknown phenotypes under natural or artificial selection. This study is, therefore, aiming to reveal and identify unique selection signatures in the genome and the genes under selection in Ethiopian indigenous goat populations using whole genome resequencing data.

## Materials and Methods

### Animals and Sampling

**Figure 1:**
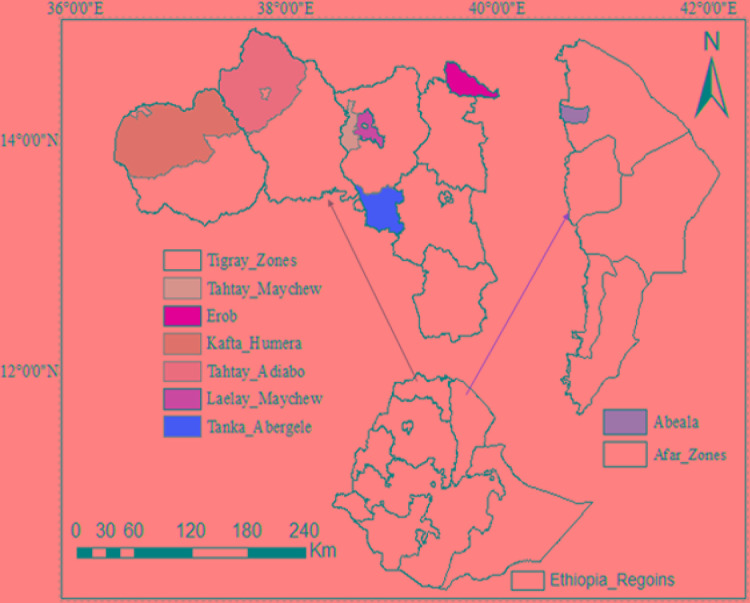
Geographic distribution and sample collection site of the five indigenous goat populations.

We sampled a total of 44 goat from five Ethiopian indigenous goat populations (Aberegalle (n = 11), Afar (n = 5), Begait (n = 11), Central highland (n = 12) and Meafure (n = 5). The samples were collected from two provinces (Afar and Tigray) as indicated in (**Figure 1**). All samples were collected from representative unrelated individual animals. To ensuring the representative sampling of the animals, livestock experts, herdsmen and owners of the animals were consulted. Following this, Afar (AF) goat samples were collected from two villages and five flocks. Aberegelle (AB) goat samples were obtained from one research station and two districts while, the Begait (BG) goat samples were obtained from two villages, one research station, and one university farm. Central highland (CH) goat samples were obtained from 4 villages. Meafure (MR) goat samples were collected from 3 local areas. Among these five goat populations, Begait and Abergelle goat populations are highly selected for different purposes (meat and milk production). Begait goats produce a high daily milk yield (0.6 kg)), and have high productive potential since they have better early birth weight and growth rates compared to the other goat populations (Berhane and Eik, 2006). They are the most preferred breeds by livestock keepers because of their excellent twinning ability, body size, pre-weaning kid survival rate, and milk yield (Abraham, 2018), while the Abergelle goats are a dual purpose and widely distributed in areas dominated by plains and river valleys. They are stocky, compact, and well built (height at withers of 71.4 and 65 cm for males and females, respectively), with a body weight of 34 and 28 kg for males and females, respectively, and these are the key identifying physical features of the these goats (Berhane and Eik, 2006). The majority of these goats have commonly a plain coat colour (56%), with 33% patchy and 11% spotted. They have short and smooth hair (Berhane and Eik, 2006). A detailed description of the appearance, characteristics, and sample distributions of the populations is provided in (**Supplementary Table S1**). Arising from this different phenotypic characteristics and breeding histories, these two groups of animals could therefore provide appropriate genetic material to analyze genomic differences among the animals.

### DNA Extraction

For all samples, blood was obtained from veins using FTA^®^ cards following the procedure of Berry Genomics (www.berrygenomics.com). Until DNA extraction, blood samples were stored at room temperature. DNA extractions were performed at the Institute of Animal Science, Chinese Academy of Agricultural Sciences, Beijing, China, based on the manufacturer’s protocol. The library construction and whole genome resequencing was performed for 150 bp paired-end reads using the Hiseq-3000 platform (Illumina, San Diego, CA, USA).

### Whole Genome Resequence, Quality Checking and Mapping

The fastQC software (Hadfield et al., 2014) was used to perform a raw sequence data quality check. Using BWA (Li and Durbin, 2010), paired-end read data were processed to filter out the adaptors and low quality reads resulted in clean reads. After alignment to the goat reference assembly(ARS1) (Bickhart et al., 2018), variants were called using SAMtools package 0.1.2 (Li et al., 2009) and were used to convert file format from SAM to BAM. On average, 99.2% of the reads were mapped, resulting in a final average sequencing death of 16 (10× to 30×) per individual. Picard ver. 1.86 (http://picard.sourceforge.net) were used to sort BAM file and remove potential MarkDuplicates if multiple read pairs had identical external coordinates. Read pairs with the highest mapping quality were retained. Single nucleotide polymorphisms (SNPs) calling was performed using both GATK (McKenna et al., 2010) and SAMtools (Li et al., 2009) the output was further filtered using Variant Call Format (VCF) tools (Danecek et al., 2011). SNPs that did not meet the following criteria were excluded: (1) 3× < mean sequencing depth (overall included individuals) < 30×; (2) a minor allele frequency > 0.05; (3) maximum missing rate < 0.1; and (4) only bi-alleles autosomal were used for further analysis.

### Population Structure and Admixture Analyses

To determine the genetic relatedness among the study goat samples, whole-genome autosomal SNPs were extracted using PLINK v1.9 (Purcell et al., 2007). Principal component analysis (PCA) was carried out to illustrate the relationship among the populations from the genetic relationship matrix of the first two eigenvalues using the smart PCA program in the package EIGENSOFT version 6.0.1 (Patterson et al., 2006), and plotted in R package version 3.4 (Zheng et al., 2012). ADMIXTURE version.1.3.0 were used to assess the population the genome-wide genetic structure among the populations (Alexander et al., 2009). To examine the most ideal population structure, a cross-validation procedure was undertaken with hypothetical ADMIXTURE runs from K = 1 to 10.

### Genome-Wide Selective Sweep Analysis

To identify genome-wide selective sweeps, we used two populations (AB and BG as they have enough sample, better phenotypic characteristics and breeding histories) by using the two statistical approaches; population differentiation (*F_ST_*) and pooled heterozygosity (*H_P_*). Individual *F_ST_* values derived from pairwise comparisons between two breeds were then combined and averaged across all SNPs using a Linux script to obtain the transformed summary statistic (Akey et al., 2010).

First, the weighted *F_ST_* values were calculated for each SNP based on $F_{ST}=\frac{s^{2}}{p\left(1-p\right)+{\frac{s}{r}}^{2}}$ where *s*^2^ represents the sampling variance of allele frequencies between two populations, *p* represents the overall average allele frequency across populations and *r* = 2 represent the number of populations (Weir and Clark Cockerham, 1984) and using a sliding-window approach (100-kb windows with 50-kb increments) as described previously (Yang et al., 2016; Li et al., 2017). The *F_ST_* values were then Z transformed as $ZF_{ST}=\frac{F_{ST}-\mu F_{ST}}{\sigma F_{ST}}$ . To reduce false positive results, only the top 1% of windows with the highest average Z*F_ST_* value was positively selected as the candidate outliers under strong selective sweeps for each comparison.

Second, we determined the pooled heterozygosity (*H_P_*) values to scan selection signals as follows $H_{P}=\frac{2\sum n_{Maj}\sum n_{Min}}{{\left(\sum nMaj+\sum nM\text{in}\right)}^{2}}$ where Σ*nMaj* and Σ*nMin* represent the sums of the numbers of the major and minor alleles at each locus, respectively. Individual *H_P_* values were Z transformed as follows: $ZH_{P}=\frac{H_{P}-\mu H_{P}}{\sigma H_{P}}$ where *µH_P_* is the overall average heterozygosity and *σH_P_* is the standard deviation for all windows within each group. The extremely low *H_P_* scores (Z*H_P_* < -4 cut-off) were proposed to be selection signals using 100-kb sliding windows with a step size of 50 kb according to the method used previously (Rubin et al., 2010). All of the outlier windows were assigned to corresponding SNPs and genes.

Based on genome annotation, a gene was deemed to show evidence of being under candidate selection if it overlapped with an outlier genomic window based on both Z*F_ST_* and Z*H_P_* values. Finally, we retrieved the caprine gene (ARS1), Gene transfer format (GTF) file from the Ensembl genome browser (http://www.ensembl.org/) databases. Enrichment and functional annotation of the candidate genes were defined using the Enrichr program (Chen et al., 2013) with default settings on the human gene set. We further analysis the significant over-representation of GO biological processes (GO-BP), molecular function (GO-MF), cellular component (GO-CC) and KEGG-pathway. Only pathways or annotations with P < 0.05 were used. The functions of the candidate genes were consulted based on the annotations in the NCBI (http://www.ncbi.nlm.nih.gov in the PubMed, and literatures.

## Results

### Genome Sequence Mapping and SNPs Calling

After resequencing and mapping of clean reads, we identified 16.56 million SNPs and 123,577 Indels (insertions and deletions) from 44 individuals. Consequently, for each individual, 99.23% of total clean reads were mapped against the latest goat reference genome (assembly ARS1) with coverage of ∼99.88%. Accordingly, for each individual, more than 99.22% of total clean reads were mapped against the latest goat reference genome assembly (ARS1) with coverage of mapped ∼99.87% (**Table 1**). These mapped reads also generated an average sequencing depth of 16× per breed ranging (10× to 30×) fold, indicating that high-quality sequences were obtained in this study. Among the five goat populations, we identified 13,061,914 unique SNPs based on these stringent thresholds and used for detection of positive selection signature analysis. The largest number 12,229,657 of SNPs was detected in CH goat followed by the AB goat (11,137,576), which likely reflects a larger number of samples size. In contrast, the MR goat displayed the lowest number of SNPs 10, 749, 99 (**Table 2**), which might be due to low sequencing depth.

**Table 1:** Summary of mapping results read for clean read in in five goat populations.

Population	Raw read	Clean read	Mapped (%)	Genome coverage (%)
Abergelle	2,840,442,381	252,066,700	99.21	99.88
Afar	3,530,306,848	300,102,644	99.29	99.86
Begait	4,865,169,263	432,174,322	99.25	99.88
Central highland	2,677,763,758	238,323,758	99.17	99.88
Meafure	1,290,825,402	110,876,224	99.28	99.87
Merged	1,520,450,7652	1,333,543,648	99.22	99.88

**Table 2:** Summary of genome-wide SNPs in five goat populations.

Population	Sample code	n	SNPs	H_P_	Sequence depth
Abergelle	AB	12	11137576	0.341	10x
Afar	AF	5	10760581	0.331	30x
Begait	BG	11	10833847	0.340	20x
Central highland	CH	11	12229657	0.343	10x
Meafure	MR	5	10749996	0.341	10x

### Population Genetic Structure Analyses

**Figure 2:**
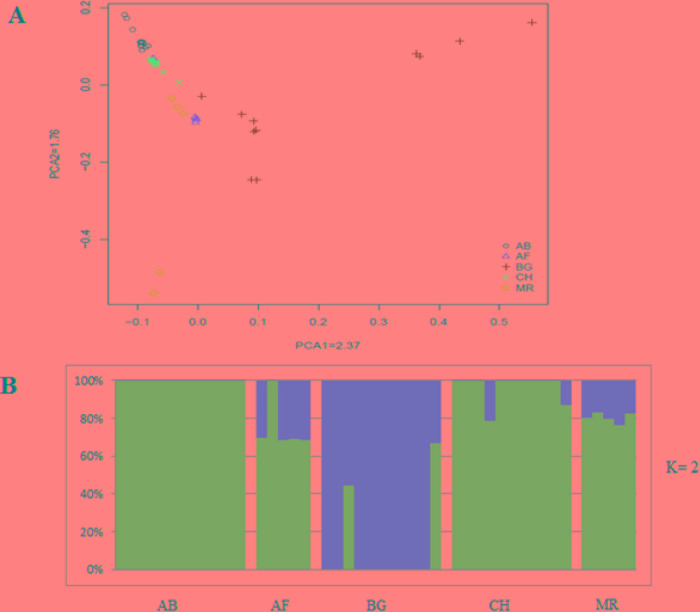
Principal component analysis five indigenous goats.

As a different approach to characterize divergence, the genetic relationship between animals was analyzed by principal component analysis (PCA). We observed closer clustering pattern between Abergelle and Central highland goat, and between Afar and Meafure goats, whereas Begait goat formed a separate cluster (**Figure 2**). This clustering pattern is consistent with the geographical locations, genetic composition and domestication history of the breeds. We also observed a clear signature of genetic admixture among all the five goat breeds. However, at K = 2, 3 and 4, an independent genetic admixture was observed in Begait goat (**Supplementary Figures 1**), which is much consistent with the PCA results. At K = 5-10, high genetic admixture was observed among all the populations (**Supplementary Figures 2**).

### Identification of Positive Selective Sweeps

For the detection of selective sweeps, we only used 2 goat populations containing 23 individuals such as Abergelle (n = 12) and Begait (n = 11). The results of PCA and Admixture, revealed that Begait goats were relatively distinct divergence compared the others (Abergelle and Central highland goats are very close relationships) goat populations.

**Figure 3:**
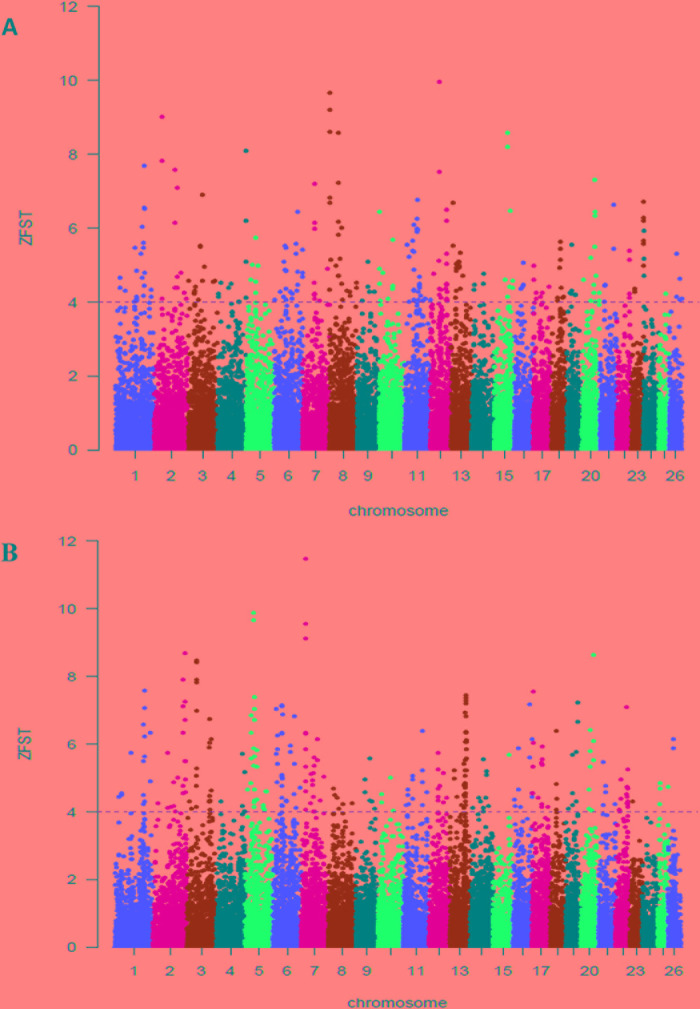
Genome-wide distributions of selection signals between Begait and Abergelle **(A)**, between Begait and Central Highland **(B)** goat populations. Manhattan plot of Z-transformed population differentiation (*F_ST_*) between any two populations for autosomal 100-kb windows 50-kb increments. The Z-value distribution plotted along goat autosomes 1–26 chromosomes.

On the basis of this results, we used selection signature to explore differences among groups of populations that are exposed to different selection pressures by calculating the average ‘Z*F_ST_*’ values between Abergelle versus Begait (**Figure 3A**) and between Begait versus Central Highland goats (**Figure 3B**). In all the comparisons, we obtained 480 outlier windows (**Supplementary Table S2**). The regions with the highest degree of differentiation were found on CHI1, 2, 3, 6, 10, and 12. In this result, *UGT2A2* was found in the selection region of Begait goat (*F_ST_* = 0.222, Z (*F_ST_*) _BG-AB_ = 6.456).

**Figure 4:**
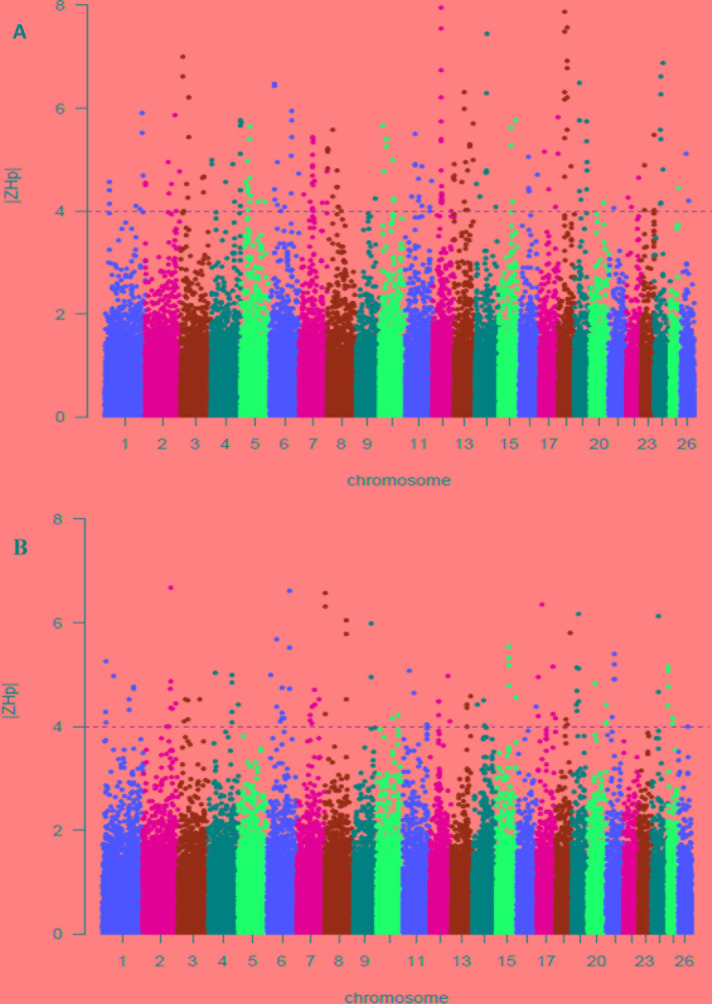
Selection sweep analyses identified putative signals in the Begait **(A)** and Abergelle **(B)** goat populations. The distributions of Z-transformed pooled heterozygosity (*H_P_)* of three populations for autosomal 100-kb windows 50-kb increments. The Z-value distribution plotted along goat autosomes 1–26 chromosomes. The dashed horizontal line indicates the cut-off (Z > 4 or Z < -4) used for extracting outliers.

From this analysis of pooled heterozygosity (*H_P_*), the overall average *H_P_* values across all the SNPs were 0.341 and 0.340 in the Abergelle, and Begait (**Table 2**), respectively. Moreover, only the lowest Z*H_P_* scores (Z*H_P_* ≤ -4) were deemed as selection regions, and were detected to be 108 and 205 outliers windows in Begait and Abergelle goats, respectively (**Supplementary Table S3**). **Figure 4A** shown the putative selection sweeps for Begait (Z (*H_P_*) _BG_) goat whereas, **Figure 4B** presented for Abergelle (Z (*H_P_*) _AB_) goat population.

### Overlap Between Candidate Selection Sweep Regions and Functional Enrichment Analyses

Putative genomic signals that overlapped between both the two approaches (high Z*F_ST_* and low Z*H_P_*) were further considered as putative selection signatures and found 11 and 5 genes for Begait and Abergelle goats (**Table 3**), respectively. Comparing their genomic locations, there is limited overlap between the different breeds showed that most of these selection signals were breed-specific, reflecting distinct phenotypic evolutions under different selection objectives or adaptations to the local environments. Within the putative selection regions identified, the lists of genes were used to perform functional analyses using Enrichr program with the default settings on the human. We identified a total of 39 GO terms (12 in biological processes, 8 in cellular component and 11 in the molecular function) and 10 KEGG pathway were annotated (**Supplementary Table S4**). The biological processes (BP) terms overrepresented were related to negative regulation of protein polymerization, cellular response to oxidative stress, regulation of histone deacetylase activity, rRNA transport, microtubule nucleation by microtubule organizing center and insulin-like growth factor receptor signaling pathway.

**Table 3:** top overlapped selection regions identified *via* Z*F_ST_* and Z*H_P_* in Begait and Abergelle goat populations associated with important traits.

Candidate Genes	Chr.	Start	End	*F_ST_*	Z*F_ST_*	*H_P_*	*Z_HP_*
*SCN9A*	2	10.63	10.64	0.208	3.563	0.080	–6.665
*CPB1*	1	11.89	11.91	0.144	3.600	0.154	–4.772
*UGT2A2*	6	85.70	85.80	0.222	6.456	0.082	–6.604
*CAMK2D*	6	12.25	12.35	0.207	5.846	0.146	–4.981
*KANK4*	3	37.65	37.75	0.238	6.626	0.164	–4.506
*RSPH6A*	18	54.40	54.50	0.187	4.617	0.114	–5.795
*NIN*	10	59.40	59.50	0.183	4.940	0.178	–4.148
*CAB39L*	12	67.65	67.75	0.242	4.406	0.146	–4.967
*SLAIN1*	12	33.20	33.30	0.132	3.941	0.031	–7.537
*TMEM117*	5	35.60	35.70	0.286	8.674	0.164	–4.293
*PNPT1*	11	37.95	38.05	0.209	6.164	0.140	–4.890
*NEK1*	8	13.00	14.00	0.150	4.249	0.126	–5.220
*PPP4R3B*	11	37.90	38.00	0.206	6.070	0.115	–5.499
*PPP1R1A*	5	25.25	25.35	0.123	3.431	0.059	–7.197
*GIGYF2*	3	83.50	84.50	0.216	3.932	0.058	–7.22
*PFKP*	13	44.55	44.65	0.209	3.795	0.149	–4.885
*ABCA13*	4	11.33	11.34	0.188	3.329	0.151	–4.828

The cellular components (CC) include; microtubule minus-end, centrosome, microtubule organizing center, cytoplasmic exosome, platelet alpha granule membrane, calcium channel complex, cytoplasmic microtubule and cytoplasmic stress granule. Whereas, the molecular functions (MF) were related to cuprous ion binding, sodium channel inhibitor activity, poly (G) binding, phosphofructokinase activity, protein serine/threonine phosphatase inhibitor activity, syntaxin-1 binding, metallocarboxypeptidase activity, cadherin binding and TPase activity, coupled to transmembrane movement of substances. The Long-term potentiation, RNA degradation, Glucagon signaling pathway, AMPK signaling pathway, Adrenergic signaling in cardiomyocytes, Axon guidance, and Galactose metabolism pathways were among the enriched in the KEGG pathways. In this study, we mainly focus on and discuss the genes and pathways that putatively contribute to the economic important traits.

## Discussion

In the present study, we performed a whole-genome resequencing of 44 goats from 5 Ethiopian goat populations. Ethiopian indigenous goat breeds are phenotypically categorized in o 14 main populations (Getinet, 2016). However, many of them are named after the community, which keeps the population, or according to geographical localities they inhabit, and the true genetic relationship between the major populations is not yet well known or documented. Among those indigenous goats, the Begait goats are Nubian type of goat which is widely used as source of meat and milk in the North-west part of Tigray, Ethiopia. Nubian goats came from Asia. It is assumed that the first wave of goats entered Ethiopia from the north between 2000 and 3000 B.C (Zeder and Hesse, 2000). In our study, the Begait goat showed high genetic variation compared to Afar, Abergelle, Central highland and Meafure goats, this might be due to artificial/natural selection. Understanding how natural selection shapes genetic variation in populations is of paramount importance in evolutionary biology (Brito et al., 2015).

This study is, therefore, the first whole genome resequencing to the breed under selection characterize genomic polymorphisms among the indigenous goat populations. The availability of high-throughput genotyping, sequencing technologies and analysis of large datasets offer great opportunities to better understand the mechanisms that underlie traits that have been exposed to intensive natural selection forces in both wild and domestic species (Lai et al., 2016; Li et al., 2017). In recent years, the selection of signature studies have become increasingly popular because they offer a complementary strategy for whole genome resequencing—to scan the goat genome for positions that may have been targeted traits of interest and selection of loci for breeding programs—and are useful for the annotation of significant functional genomic regions and thus help to link phenotypes to gene function, which could be of biotechnological relevance. We did find overlap between the selected regions identified with the *F_ST_* and *H_P_* approaches. Comparing their genomic locations showed that most of these selection signals were breed-specific, reflecting distinct phenotypic evolutions under different selection objectives or adaptations to the local environments. Based on the genetic divergence and patterns of admixture among the studied goat populations, here we only used 3 populations containing 34 individuals for the detection of selection signature. Therefore, in this study we focused the analysis on genes that are known to be associated with reproduction, body weight, and fatty acids traits.

### Candidate Genes Associated With Reproduction Traits

Reproduction traits are economic importance traits in livestock breeding in general (Laske et al., 2012) and in the goat industry in particular (Lai et al., 2016). It appears to be controlled by multiple genes and factors, (Hanrahan et al., 2004) including ovarian follicular development, embryogenesis, embryo implantation, oocyte maturation, ovulation, fertilization, and uterine receptivity.

Interestingly, in Begait goats, we identified several genes (*CAMK2D, KANK4 NIN, RSPH6A* and *UGT2A2*). The *CAMK2D* gene is involved in protein serine/threonine kinase activity (GO: 0004674). *CAMK2D* gene play an important role in bovine embryo development process (Adams et al., 2011). The gene *NIN* contain a highly differentiated region (*F_ST_* = 0.183, Z*F_ST_* = 4.940) and a low heterozygosity (*H_P_* = 0.178, Z*H_P_* = - 4.148) at 59.40 - 59.50 Mb on chromosome 10, which is involved in early embryo development. Ninein (Nin) gene is the centrosome-associated proteins which play significant roles in microtubule stability (Kowanda et al., 2016). The *RSPH6A* gene is identified in CHI18 (54.40 - 54.50 Mb) and is associated with fertility (Abbasi et al., 2018). Similarly, the region between 85.70 and 85.80 Mb on chromosome 6 showed high differentiation (*F_ST_* = 0.222, Z*F_ST_* = 6.456) and a low heterozygosity (*H_P_* *=* 0.082, Z*H_P_* = -6.604). According to genome annotation, this sweep region harbored gene *UGT2A2*, which is involved in capable of acting on estrogens. Estrogens and estrogen-like substances are widely distributed in plants and animals (Gruber et al., 2002). Estrogens are one class of steroid hormones that includes estrone, estradiol (E2), and estriol (Yaşar et al., 2017). Estradiol, the most potent estrogen hormone in the circulation, is involved in a wide variety of vital physio-logical functions that range from the development and maintenance of reproductive organs to the regulation of cardiovascular, musculoskeletal, immune, and central nervous system homeostasis (Yaşar et al., 2017).

In the genome of Abergelle goat, we identified genetic regions with the highest *F_ST_* values (*F_ST_* = 0.132, Z*F_ST_* = 3.941) and lowest *H_P_* values (*H_P_* = 0.031, Z*H_P_* = -7.537) from 33.20 - 33.30 Mb on chromosome 12 contained strongest candidate genes (*SLAIN1*). *SLAIN1* gene play role in the development of the nervous system and morphogenesis of several embryonic structures. *SLAIN1* was expressed at the stem cell (Hirst et al., 2006). Moreover, in the same breed at the starting region (13.00 – 14.00 Mb) of chromosome 8 had the differentiation values (*F_ST_* = 0.150, Z*F_ST_* = 4.249) and *H_P_* scores (*H_P_* = 0.126, Z*H_P_* = -5.220) encompassing *NEK1* gene. *NEK1* (NIMA-related kinase 1) gene is a serine/threonine and tyrosine kinase and is essential roles during meiosis. *NEK1* gene has been implicated in in ciliogenesis (White and Quarmby, 2008) and DNA damage response (Higelin et al., 2018). The gene *NEK1* is highly expressed in mouse germ cells (Chen et al., 2011).

### Candidate Genes Associated With Body Weight Traits

Growth rate and body weight are the most economically important traits in livestock that are specialized for meat production. Most of the time, tropical breeds tend to have small body weight/size and growth rate as compared with temperate breeds (Mwacharo et al., 2017). Body weight is one of the most important traits for meat type animals that can be measured at birth or at other life stages. Hence, natural selection may have left genomic footprints in the underlying genes involved the production traits of the Ethiopian goats. In the Begait goat, we found *SCN9A* gene at 10.63 - 10.64 Mb on CHI 2 with high *F_ST_* values (*F_ST_* = 0.208, Z*F_ST_* = 3.563) and low *H_P_* scores (*H_P_* = 0.080, *Z_HP_* = -6.665). Previous study indicated the *SCN9A* gene is highly associated with body size and weight in cattle (Wang et al., 2019).

In the Abergelle goat breed, we identified 3 genes (*GIGYF2* and *PPP1R1A*) at *F_ST_* and low *H_P_*, which are associated with various production traits. *GIGYF2* gene associated with growth trait at considerable impacts on lamb survivability and growth performance traits in sheep (Ghasemi et al., 2019). *PPP1R1A* was significantly associated gene with body mass index (Seo et al., 2016) and it is also known that positively correlated with insulin secretion in human (Taneera et al., 2014) and it also affects body mass index (BMI) in humans (Dina et al., 2007).

### Candidate Genes Related to Fatty Acids

It is known that several candidate genes in the putative selection regions are involved in regulating fatty acids in mammals. Fatty acids are required by daily normal metabolism, and is an important trait contributing to meat quality (Zhu et al., 2017). Previous studies have been conducted to examine fatty acids for various goat breeds (Banskalieva et al., 2000). Interestingly, in the Begait goat, we identified *CAB39L* gene at high *F_ST_* values (*F_ST_* = 0.207, Z*F_ST_* = 3.625) and low *H_P_* scores (*H_P_* = 0.175, *ZH_P_* = -4.218) located at 81.45-81.55Mb on CHI 10, and this gene play role in cell adhesion receptors and growth factor receptors. Growth factor–induced cell proliferation, adhesion, and migration in cultured cell models often depend on specific integrin (Eliceiri, 2001). Other candidate genes identified include *TMEM117* (transmembrane protein 11) located on CHI 5 (*F_ST_* = 0.286, Z*F_ST_* = 8.674) and (*H_P_* = 0.164, *ZH_P_* = -4.293) at 35.60 - 35.70Mb in Abergelle goats. *TMEM117* gene has strongly associated with saturated fatty acids composition in Simmental cattle (Zhu et al., 2017).

### Candidate Genes Related to Disease

Moreover, Metabolic syndrome (MetS) is a set of complex disorders characterized by central obesity, high blood pressure, hypercholesterolemia, hypertriglyceridemia, decreased high-density lipoprotein cholesterol, and glucose intolerance (Lan et al., 2015). In Abergelle goats, we identified two genes, *PPP4R3B* (37.90 - 38.00 Mb) and *PNPT1* (37.95 - 38.05 Mb) on CHI11. *PPP4R3B* is involved in gluconeogenesis, lipid metabolism and facilitates the uncovering of molecular mechanisms and in the identification of novel therapeutic targets for the disease (Kristiansson et al., 2012). *PNPT1* gene was reported to be implicated with multiple metabolic RNA processing-related genes in pig (Park et al., 2010). Reduction of *PNPT1* levels results in impaired mitochondrial processing and accumulation of large polycistronic transcripts, possibly due to its connection to the import of RNase P RNA into the mitochondria (Pellegrina et al., 2017).

## Conclusion

Using a whole genome resequence data, we studied for the first-time selection signals in Ethiopian indigenous goat populations that may have left genomic footprints in the underlying genes involved in important traits. This genomic data generated several potential candidate genes related to body weight, reproduction and fatty acid traits in goats, reflecting phenotypic evolution under different selection signals and adaptation to the local environments. Furthermore, these results provide a basis for further investigation on the genomic characteristics of Ethiopian goat populations for complex traits.

## Data Availability Statement

Whole genome resequencing data of 44 animals representing five Ethiopian goat populations after variant calls and genotype calls used in this paper are deposited and available at (http://bigd.big.ac.cn/gvm/getProjectDetail?project=GVM000049; Accession number: GVM000049).

## Ethics Statement

Whole blood sample from the Ethiopian indigenous goat were collected after agreement from the local authorities and owners of the animals. No further specific permissions were required from the Ethics Committee of the Mekelle University, Ethiopia at the time of the sampling.

## Author Contributions

LJ, YM and HB designed the study. LJ and YM generated the genome data. YL and HB participated in the data analysis. HB, BG and GG participated in DNA extraction, HB and BG sampled individuals. HB drafted the manuscript with the inputs from all authors. All the authors revised and approved the manuscript.

## Funding

This work was financial supported by the National Natural Science Foundation of China (31601910 and U1603232).

## Conflict of Interest

The authors declare that the research was conducted in the absence of any commercial or financial relationships that could be construed as a potential conflict of interest.

## Supplementary Material

Supplementary Figure 1 Population structure analysis (K=2-4).

Supplementary Figure 2 Population structure analysis (K=5-10).

Supplementary Table S1 Ethiopian indigenous goat population information. The characteristics, including physical characteristics and distributions.

Supplementary Table S2 List of genes in selected overlapping regions by top 1% of windows with the highest average ZFST value for Aberegelle goat population.

Supplementary Table S3 List of gene in the sellected lowest score (ZHP < -4 cut off ) for Begait goat population.

Supplementary Table S4 Gene Ontology analysis.
