# Supplementary figures and images for: Whole Genome Resequencing Reveals Selection Signatures Associated With Important Traits in Ethiopian Indigenous Goat Populations
Source: Front Genet. 2019 Nov 28;10:1190. doi: 10.3389/fgene.2019.01190 (PMC6892828; doi:10.3389/fgene.2019.01190)

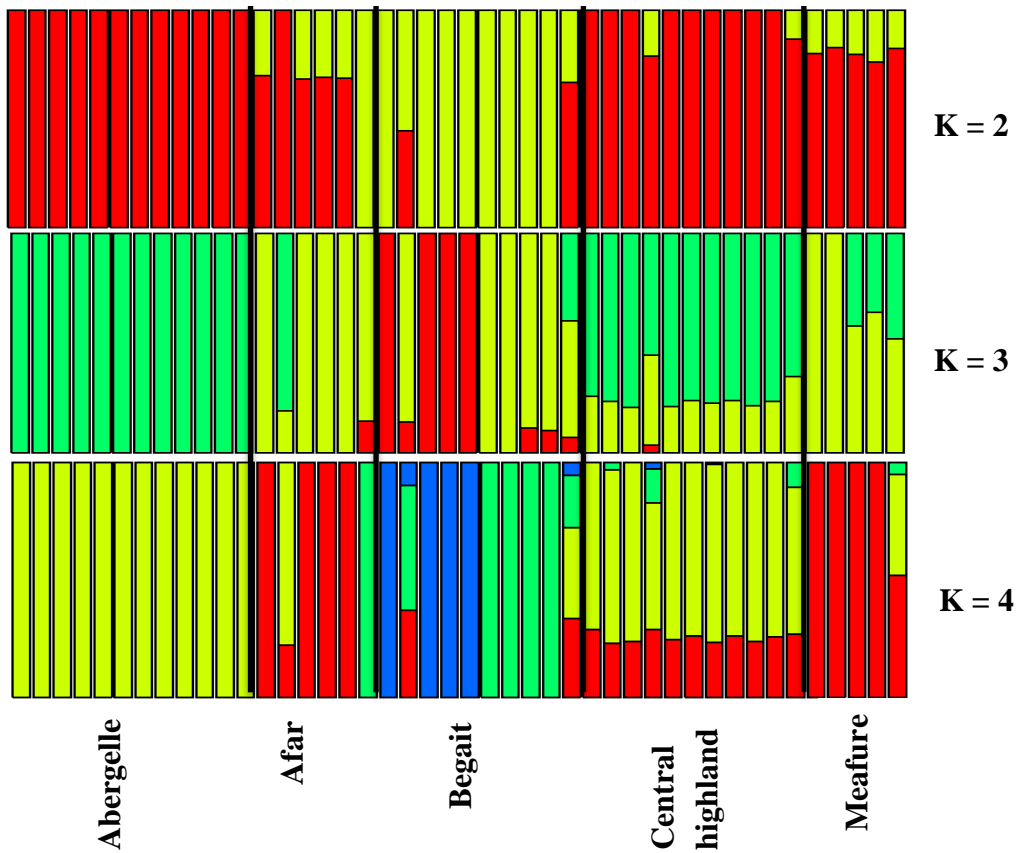

Supplement: Supplementary Figure 1 — Population structure analysis (K=2-4). [file DataSheet_1.zip › Supplementary Figure 1.pdf]

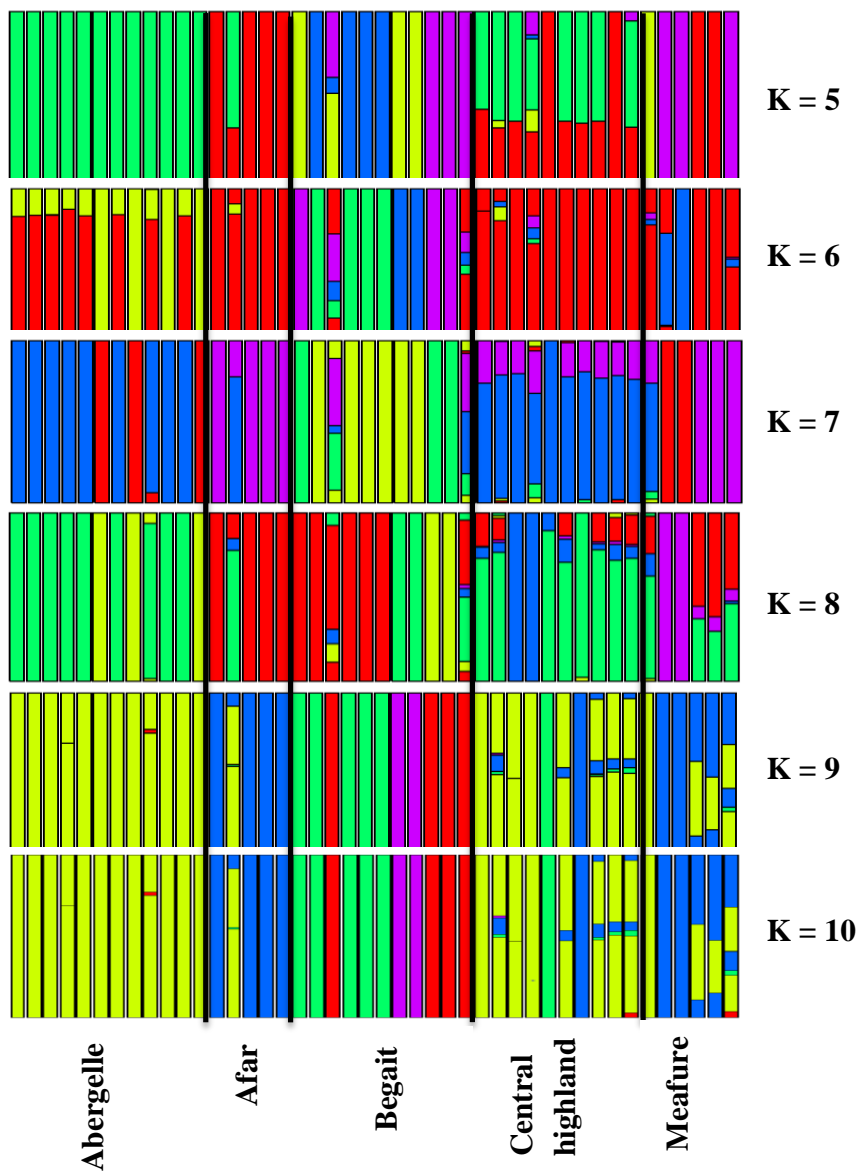

Supplement: Supplementary Figure 1 — Population structure analysis (K=2-4). [file DataSheet_1.zip › Supplementary Figure 2.pdf]
